# Supplementary material for: The discriminative power of the ReproQ: a client experience questionnaire in maternity care
Source: PeerJ. 2019 Nov 27;7:e7575. doi: 10.7717/peerj.7575 (PMC6884994; doi:10.7717/peerj.7575)
Supplement: Supplemental Information 3 [file peerj-07-7575-s003.docx]

# Code of the article ‘The discriminative power of the ReproQ: a client experience questionnaire in maternity care’

## Multilevel analysis - performed in R

**Pregnancy** - without case mix correction

[1] " respect, tweede helft zwangerschap "

Linear mixed model fit by REML ['lmerMod']

Formula: RQ_R_Dom_B ~ (1 | VSVnr)

Data: reproq

REML criterion at convergence: 1791.8

Scaled residuals:

Min 1Q Median 3Q Max

-9.7328 -0.1156 0.4380 0.4781 0.6037

Random effects:

Groups Name Variance Std.Dev.

VSVnr (Intercept) 0.0004017 0.02004

Residual 0.0771874 0.27783

Number of obs: 6371, groups: VSVnr, 42

Fixed effects:

Estimate Std. Error t value

(Intercept) 3.867812 0.004819 802.7

[1] 0.0004016787

[1] 0.07718744

Linear mixed model fit by REML ['lmerMod']

Formula: RQ_R_Dom_B ~ (1 | VSVnr)

Data: reproq

REML criterion at convergence: 1791.8

Scaled residuals:

Min 1Q Median 3Q Max

-9.7328 -0.1156 0.4380 0.4781 0.6037

Random effects:

Groups Name Variance Std.Dev.

VSVnr (Intercept) 0.0004017 0.02004

Residual 0.0771874 0.27783

Number of obs: 6371, groups: VSVnr, 42

Fixed effects:

Estimate Std. Error t value

(Intercept) 3.867812 0.004819 802.7

[1] 0.0004016787

[1] 0.07718744

[1] "____"

**Pregnancy** - with case mix correction

[1] " respect, tweede helft zwangerschap "

Linear mixed model fit by REML t-tests use Satterthwaite approximations to degrees of freedom [lmerMod]

Formula: RQ_R_Dom_B ~ SD_Lft_M_Cat4 + SD_Opl_M_Cat3 + OV_Gez_M_4 + (1 | VSVnr)

Data: reproq

REML criterion at convergence: 1465.6

Scaled residuals:

Min 1Q Median 3Q Max

-9.9523 -0.0674 0.3643 0.4852 1.1988

Random effects:

Groups Name Variance Std.Dev.

VSVnr (Intercept) 0.0002829 0.01682

Residual 0.0734406 0.27100

Number of obs: 6091, groups: VSVnr, 42

Fixed effects:

Estimate Std. Error df t value Pr(>|t|)

(Intercept) 3.889e+00 7.776e-03 3.110e+02 500.135 < 2e-16 ***

SD_Lft_M_Cat4 24 -4.955e-02 1.589e-02 6.075e+03 -3.119 0.00182 **

SD_Lft_M_Cat4 25-29 -1.012e-02 8.330e-03 6.074e+03 -1.215 0.22423

SD_Lft_M_Cat4 35 6.582e-04 9.508e-03 6.079e+03 0.069 0.94481

SD_Lft_M_Cat4 missing -4.956e-02 2.727e-02 6.073e+03 -1.818 0.06917 .

SD_Opl_M_Cat3 laag 2.249e-02 1.511e-02 6.066e+03 1.489 0.13656

SD_Opl_M_Cat3 middel 3.232e-02 8.004e-03 5.788e+03 4.038 5.45e-05 ***

SD_Opl_M_Cat3 missing 1.729e-02 2.113e-02 6.075e+03 0.818 0.41327

OV_Gez_M_4 uitstekend 1.984e-02 9.500e-03 6.078e+03 2.088 0.03681 *

OV_Gez_M_4 goed -5.607e-02 8.100e-03 6.080e+03 -6.922 4.91e-12 ***

OV_Gez_M_4 matig -1.695e-01 1.674e-02 6.075e+03 -10.126 < 2e-16 ***

---

Signif. codes: 0 ‘***’ 0.001 ‘**’ 0.01 ‘*’ 0.05 ‘.’ 0.1 ‘ ’ 1

Correlation of Fixed Effects:

(Intr) SD_L_M_C4 SD_L_M_C42 SD_L_M_C4 SD_Lf_M_C4 SD_Opl_M_Ct3l SD_Opl_M_Ct3md

SD_L_M_C42 -0.166

SD_L_M_C425 -0.423 0.259

SD_L_M_C43 -0.387 0.192 0.370

SD_Lft_M_C4 -0.114 0.087 0.140 0.114

SD_Opl_M_Ct3l -0.129 -0.169 -0.064 0.010 -0.051

SD_Opl_M_Ct3md -0.260 -0.215 -0.130 0.007 -0.062 0.227

SD_Opl_M_Ct3ms -0.109 -0.026 -0.022 0.002 -0.018 0.073 0.133

OV_Gz_M_4ts -0.414 -0.002 -0.004 -0.014 -0.016 -0.005 0.015

OV_Gz_M_4gd -0.456 0.002 0.013 -0.037 -0.006 -0.056 -0.079

OV_Gz_M_4mt -0.213 -0.030 0.007 -0.008 -0.002 -0.065 -0.057

SD_Opl_M_Ct3ms OV_Gz_M_4t OV_Gz_M_4g

SD_L_M_C42

SD_L_M_C425

SD_L_M_C43

SD_Lft_M_C4

SD_Opl_M_Ct3l

SD_Opl_M_Ct3md

SD_Opl_M_Ct3ms

OV_Gz_M_4ts -0.001

OV_Gz_M_4gd -0.022 0.401

OV_Gz_M_4mt -0.010 0.192 0.237

[1] 0.0002829407

[1] 0.07344057

[1] 0.003837862

[1] "____"

**Birth and postnatal period – without case mix correction**

[1] " Dignity, birth "

Linear mixed model fit by REML ['lmerMod']

Formula: RQ_R_Dom_A ~ (1 | VSVnr)

Data: reproq

REML criterion at convergence: 6701.8

Scaled residuals:

Min 1Q Median 3Q Max

-8.2131 0. 0.0028 0.4872 0.5235 0.5594

Random effects:

Groups Name Variance Std.Dev.

VSVnr (Intercept) 0.0002815 0.01678

Residual 0.1179841 0.34349

Number of obs: 9528, groups: VSVnr, 55

Fixed effects:

Estimate Std. Error t value

(Intercept) 3.823696 0.004417 865.7

[1] 0.0002814849

[1] 0.1179841

[1] 0.002380108

[1] "____”

[1] " Dignity, Postnatal period "

Linear mixed model fit by REML ['lmerMod']

Formula: RQ_R_Dom_B ~ (1 | VSVnr)

Data: reproq

REML criterion at convergence: 7337.4

Scaled residuals:

Min 1Q Median 3Q Max

-7.8143 -0.0287 0.4440 0.5538 0.6543

Random effects:

Groups Name Variance Std.Dev.

VSVnr (Intercept) 0.00106 0.03256

Residual 0.12617 0.35521

Number of obs: 9489, groups: VSVnr, 55

Fixed effects:

Estimate Std. Error t value

(Intercept) 3.808899 0.006061 628.4

[1] 0.001059885

[1] 0.1261739

[1] 0.008330219

[1] "____"

**Birth and postnatal period – with case mix correction**

[1] "Casemix modellen - regel 1= variantie instellingen, regel 2= varientie residual, regel 3=ICC" [1] "____"

[1] " Dignity, birth"

Linear mixed model fit by REML t-tests use Satterthwaite approximations to degrees of freedom [lmerMod]

Formula: RQ_R_Dom_A ~ SD_Lft_M_Cat4 + SD_Opl_M_Cat3 + OV_Gez_M_4 + (1 | VSVnr)

Data: reproq

REML criterion at convergence: 6195.3

Scaled residuals:

Min 1Q Median 3Q Max

-8.6103 -0.1066 0.3458 0.5262 1.3253

Random effects:

Groups Name Variance Std.Dev.

VSVnr (Intercept) 0.0002468 0.01571

Residual 0.1146282 0.33857

Number of obs: 9112, groups: VSVnr, 55

Fixed effects:

Estimate Std. df t value Pr(>|t|)

(Intercept) 3.835e+00 7.791e-03 5.060e+02 492.170 < 2e-16 ***

SD_Lft_M_Cat4 24 -7.697e-02 1.679e-9.090e+03 -4.585 4.60e-06 ***

SD_Lft_M_Cat4 25-29 -1.286e-02 8.695e-03 9.076e+03 -1.479 0.139

SD_Lft_M_Cat4 35 -3.124e-04 9.189e-03 9.082e+03 -0.034 0.973

SD_Opl_M_Cat3laag -1.008e-02 1.381e-02 9.027e+03 -0.730 0.466

SD_Opl_M_Cat3middel 3.623e-02 7.956e-03 8.531e+03 4.553 5.35e-06 ***

OV_Gez_M_4uitstekend 5.639e-02 9.092e-03 9.103e+03 6.202 5.82e-10 ***

OV_Gez_M_4goed -7.004e-02 8.490e-03 9.103e+03 -8.250 2.22e-16 ***

OV_Gez_M_4matig -2.060e-01 2.004e-02 9.093e+03 -10.281 < 2e-16 ***

---

Signif. codes: 0 ‘***’ 0.001 ‘**’ 0.01 ‘*’ 0.05 ‘.’ 0.1 ‘ ’ 1

Correlation of Fixed Effects:

(Intr) SD_L_M_C4¤ SD_L_M_C4¤ SD_L_M_C4¤ SD_L_M_C42 SD_L_M_C42 SD_L_M_C42 SD_L_M_C4¥ SD_L_M_C4¥ SD_L_M_C4¥ SD_Opl_M_Ct3l SD_Opl_M_Ct3l SD_Opl_M_Ct3l SD_Opl_M_Ct3m SD_Opl_M_Ct3m SD_Opl_M_Ct3m OV_Gz_M_4t OV_Gz_M_4t OV_Gz_M_4t

SD_L_M_C4 2 -0.148

SD_L_M_C425 -0.380 0.239

SD_L_M_C4 3 -0.406 0.187 0.363

SD_Opl_M_Ct3l -0.144 -0.152 -0.105 -0.008

SD_Opl_M_Ct3m -0.295 -0.194 -0.151 0.023 0.257

OV_Gz_M_4ts -0.462 -0.007 -0.016 -0.002 0.009 0.018

OV_Gz_M_4gd -0.460 -0.004 0.023 -0.029 -0.083 -0.075 0.423

OV_Gz_M_4mt -0.178 0.001 -0.012 -0.024 -0.056 -0.047 0.179

OV_Gz_M_4g SD_L_M_C4¤2 SD_L_M_C425 SD_L_M_C4¥3

SD_Opl_M_Ct3l SD_Opl_M_Ct3m OV_Gz_M_4ts OV_Gz_M_4gd OV_Gz_M_4mt 0.200

[1] 0.0002467927

[1] 0.1146282

[1] 0.002148359

[1] "____"

[1] " Dignity, Postnatal period "

Linear mixed model fit by REML t-tests use Satterthwaite approximations to degrees of freedom [lmerMod]

Formula: RQ_R_Dom_B ~ SD_Lft_M_Cat4 + SD_Opl_M_Cat3 + OV_Gez_M_4 + (1 VSVnr)

Data: reproq

REML criterion at convergence: 6765.3

Scaled residuals:

Min 1Q Median 3Q Max

-7.5025 -0.0911 0.3726 0.5500 1.1585

Random effects:

Groups Name Variance Std.Dev.

VSVnr (Intercept) 0.001142 0.03379

Residual 0.121998 0.34928

Number of obs: 9075, groups: VSVnr, 55

Fixed effects: Estimate Std. Error df t value Pr(>|t|)

(Intercept) 3.817e+00 9.120e-03 2.200e+02 418.517 < 2e-16 ***

SD_Lft_M_Cat4â‰¤24 -3.920e-02 1.742e-02 9.066e+03 -2.250 0.0245 *

SD_Lft_M_Cat425-29 -6.968e-03 9.010e-03 9.066e+03 -0.773 0.4393

SD_Lft_M_Cat4â‰¥35 -2.269e-03 9.520e-03 9.066e+03 -0.238 0.8116

SD_Opl_M_Cat3laag 1.894e-02 1.440e-02 9.054e+03 1.315 0.1884

SD_Opl_M_Cat3middel 3.238e-02 8.277e-03 8.905e+03 3.912 9.21e-05 ***

OV_Gez_M_4uitstekend 5.071e-02 9.402e-03 9.055e+03 5.394 7.07e-08 ***

OV_Gez_M_4goed -6.806e-02 8.792e-03 9.056e+03 -7.741 1.11e-14 ***

OV_Gez_M_4matig -1.781e-01 2.069e-02 9.041e+03 -8.608 < 2e-16 ***

---

Signif. codes: 0 ‘***’ 0.001 ‘**’ 0.01 ‘*’ 0.05 ‘.’ 0.1 ‘ ’ 1

Correlation of Fixed Effects:

(Intr) SD_L_M_C4¤ SD_L_M_C42 SD_L_M_C4¥ SD_Opl_M_Ct3l SD_Opl_M_Ct3m OV_Gz_M_4t

SD_L_M_C4¤2 -0.134

SD_L_M_C425 -0.337 0.239

SD_L_M_C4¥3 -0.360 0.186 0.361

SD_Opl_M_Ct3l -0.127 -0.151 -0.102 -0.011

SD_Opl_M_Ct3m -0.259 -0.192 -0.148 0.019 0.260

OV_Gz_M_4ts -0.407 -0.006 -0.015 0.000 0.008 0.017

OV_Gz_M_4gd -0.406 -0.005 0.024 -0.028 -0.081 -0.073 0.422

OV_Gz_M_4mt -0.157 0.000 -0.014 -0.025 -0.055 -0.046 0.180

OV_Gz_M_4g SD_L_M_C4¤2 SD_L_M_C425 SD_L_M_C4¥3 SD_Opl_M_Ct3l SD_Opl_M_Ct3m OV_Gz_M_4ts OV_Gz_M_4gd OV_Gz_M_4mt 0.200

[1] 0.001141764

[1] 0.1219984

[1] 0.009272069

[1] "____"

## Discriminative power - performed in SPSS

**Discriminative power based on statistics**

These results are based on the caterpillar plots made in R.

**Discriminative power based on relevance**

*Calculating the MID*

NUMERIC OV_All_M_Cat5 (F2.0).

COMPUTE OV_All_M_Cat5 =-999.

IF (OV_All_S=1 OR OV_All_S=2 OR OV_All_S=3 OR OV_All_S=4 OR OV_All_S=5 OR OV_All_S=6) OV_All_M_Cat5 =1.

IF (OV_All_S=7) OV_All_M_Cat5 =2.

IF (OV_All_S=8) OV_All_M_Cat5 =3.

IF (OV_All_S=9) OV_All_M_Cat5 =4.

IF (OV_All_S=10) OV_All_M_Cat5 =5.

IF (OV_All_S=999) OV_All_M_Cat5 =999.

VARIABLE LABELS OV_All_M_Cat5 'overall cijfer - Missing'.

VALUE LABELS OV_All_M_Cat5

1 '≤6'

2 '7'

3 '8'

4 '9'

5 '10'

999 'missing'.

MISSING VALUES OV_All_M_Cat5 (999).

FREQUENCIES OV_All_M_Cat5.

* STAP 1: Spliting the mean domain and summary scores for the 5 subgroups of the overall rating.

FREQUENCIES OV_All_M_Cat5.

SORT CASES OV_All_M_Cat5 (A).

SPLIT FILE BY OV_All_M_Cat5.

FREQUENCIES VARIABLES= RQ_R_Dom_A RQ_A_Dom_A RQ_P_Dom_A RQ_C_Dom_A RQ_T_Dom_A RQ_S_Dom_A RQ_F_Dom_A RQ_K_Dom_A RQ_PS_Dom_A RQ_ST_Dom_A RQ_EvT_A

/STATISTICS=MEAN STDDEV MEDIAN

/NTILES 4

/ORDER=ANALYSIS.

FREQUENCIES VARIABLES= RQ_R_Dom_B RQ_A_Dom_B RQ_P_Dom_B RQ_C_Dom_B RQ_T_Dom_B RQ_S_Dom_B RQ_F_Dom_B RQ_K_Dom_B RQ_PS_Dom_B RQ_ST_Dom_B RQ_EvT_B

/STATISTICS=MEAN STDDEV MEDIAN

/NTILES 4

/ORDER=ANALYSIS.

SPLIT FILE OFF.

* STAP 2: Calculating standard error of the mean.

DESCRIPTIVES VARIABLES=RQ_R_Dom_A RQ_A_Dom_A RQ_P_Dom_A RQ_C_Dom_A RQ_T_Dom_A RQ_S_Dom_A RQ_F_Dom_A

RQ_K_Dom_A RQ_PS_Dom_A RQ_ST_Dom_A RQ_EvT_A

/STATISTICS=MEAN STDDEV MIN MAX SEMEAN.

*Determining the ReproQ’s discriminative power*

* STAP 1: maximum score of the perinatal units

FREQUENCIES

RQ_EvT_B

RQ_PS_Dom_B RQ_ST_Dom_B

RQ_R_Dom_B RQ_A_Dom_B RQ_P_Dom_B RQ_C_Dom_B RQ_T_Dom_B RQ_S_Dom_B RQ_F_Dom_B RQ_K_Dom_B

/STATISTICS MEAN MEDIAN MINIMUM MAXIMUM.

SORT CASES BY VSVnr (A).

SPLIT FILE BY VSVnr.

FREQUENCIES

RQ_EvT_B

RQ_PS_Dom_B RQ_ST_Dom_B

RQ_R_Dom_B RQ_A_Dom_B RQ_P_Dom_B RQ_C_Dom_B RQ_T_Dom_B RQ_S_Dom_B RQ_F_Dom_B RQ_K_Dom_B

/STATISTICS MEAN .

SPLIT FILE OFF.

* STAP 2: Admiting the MID as variable.

COMPUTE RQ_TOT_MID_ZS = 0.11 .

COMPUTE RQ_PS_MID_ZS = 0.09 .

COMPUTE RQ_ST_MID_ZS = 0.12 .

COMPUTE RQ_Res_MID_ZS = 0.07 .

COMPUTE RQ_Aut_MID_ZS = 0.11 .

COMPUTE RQ_Pri_MID_ZS = 0.09 .

COMPUTE RQ_Com_MID_ZS = 0.11 .

COMPUTE RQ_Tyd_MID_ZS = 0.11 .

COMPUTE RQ_Soc_MID_ZS = 0.09 .

COMPUTE RQ_Fac_MID_ZS = 0.08 .

COMPUTE RQ_Keu_MID_ZS = 0.19 .

* STAP 3C: Admitting the mean score of the P90 - P100 as variable.

COMPUTE RQ_TOT_ZS_D90 = 3.796175.

COMPUTE RQ_PS_ZS_D90 = 3.806275 .

COMPUTE RQ_ST_ZS_D90 = 3.7922 .

COMPUTE RQ_Res_ZS_D90 = 3.91195 .

COMPUTE RQ_Aut_ZS_D90 = 3.750975 .

COMPUTE RQ_Pri_ZS_D90 = 3.813694125 .

COMPUTE RQ_Com_ZS_D90 = 3.802525 .

COMPUTE RQ_Tyd_ZS_D90 = 3.7661 .

COMPUTE RQ_Soc_ZS_D90 = 3.837875 .

COMPUTE RQ_Fac_ZS_D90 = 3.88975 .

COMPUTE RQ_Keu_ZS_D90 = 3.74155 .

EXECUTE.

* STAP 4C:Determining of the difference in the score of the perinatal units and the Delta 90-score is equal or larger than the MID.

NUMERIC RQ_TOT_VSV_ZS_D90 (F2.0).

COMPUTE RQ_TOT_VSV_ZS_D90 =$SYSMIS.

IF ((RQ_TOT_ZS_D90-RQ_Tot_ZS_B) >=RQ_TOT_MID_ZS) RQ_TOT_VSV_ZS_D90 =2.

IF ((RQ_TOT_ZS_D90-RQ_Tot_ZS_B) <RQ_TOT_MID_ZS) RQ_TOT_VSV_ZS_D90 =1.

VARIABLE LABELS RQ_TOT_VSV_ZS_D90 'Relevant verschil tussen vsvs D90- totaal score'.

VALUE LABELS RQ_TOT_VSV_ZS_D90

1 'no relevant difference'

2 'relevant difference'.

FREQUENCIES RQ_TOT_VSV_ZS_D90.

NUMERIC RQ_PERS_VSV_ZS_D90 (F2.0).

COMPUTE RQ_PERS_VSV_ZS_D90 =$SYSMIS.

IF ((RQ_PS_ZS_D90-RQ_PERS_ZS_B) >=RQ_PS_MID_ZS) RQ_PERS_VSV_ZS_D90 =2.

IF ((RQ_PS_ZS_D90-RQ_PERS_ZS_B) <RQ_PS_MID_ZS) RQ_PERS_VSV_ZS_D90 =1.

VARIABLE LABELS RQ_PERS_VSV_ZS_D90 'Relevant verschil tussen vsvs D90- pers score'.

VALUE LABELS RQ_PERS_VSV_ZS_D90

1 'no relevant difference'

2 'relevant difference'.

FREQUENCIES RQ_PERS_VSV_ZS_D90.

NUMERIC RQ_SETT_VSV_ZS_D90 (F2.0).

COMPUTE RQ_SETT_VSV_ZS_D90 =$SYSMIS.

IF ((RQ_ST_ZS_D90-RQ_SETT_ZS_B) >=RQ_ST_MID_ZS) RQ_SETT_VSV_ZS_D90 =2.

IF ((RQ_ST_ZS_D90-RQ_SETT_ZS_B) <RQ_ST_MID_ZS) RQ_SETT_VSV_ZS_D90 =1.

VARIABLE LABELS RQ_SETT_VSV_ZS_D90 'Relevant verschil tussen vsvs D90- sett score'.

VALUE LABELS RQ_SETT_VSV_ZS_D90

1 'no relevant difference'

2 'relevant difference'.

FREQUENCIES RQ_SETT_VSV_ZS_D90.

NUMERIC RQ_RES_VSV_ZS_D90 (F2.0).

COMPUTE RQ_RES_VSV_ZS_D90 =$SYSMIS.

IF ((RQ_RES_ZS_D90-RQ_RES_ZS_B) >=RQ_RES_MID_ZS) RQ_RES_VSV_ZS_D90 =2.

IF ((RQ_RES_ZS_D90-RQ_RES_ZS_B) <RQ_RES_MID_ZS) RQ_RES_VSV_ZS_D90 =1.

VARIABLE LABELS RQ_RES_VSV_ZS_D90 'Relevant verschil tussen vsvs D90- res score'.

VALUE LABELS RQ_RES_VSV_ZS_D90

1 'no relevant difference'

2 'relevant difference'.

FREQUENCIES RQ_RES_VSV_ZS_D90.

NUMERIC RQ_Aut_VSV_ZS_D90 (F2.0).

COMPUTE RQ_Aut_VSV_ZS_D90 =$SYSMIS.

IF ((RQ_Aut_ZS_D90-RQ_Aut_ZS_B) >=RQ_Aut_MID_ZS) RQ_Aut_VSV_ZS_D90 =2.

IF ((RQ_Aut_ZS_D90-RQ_Aut_ZS_B) <RQ_Aut_MID_ZS) RQ_Aut_VSV_ZS_D90 =1.

VARIABLE LABELS RQ_Aut_VSV_ZS_D90 'Relevant verschil tussen vsvs D90- aut score'.

VALUE LABELS RQ_Aut_VSV_ZS_D90

1 'no relevant difference'

2 'relevant difference'.

FREQUENCIES RQ_Aut_VSV_ZS_D90.

NUMERIC RQ_Pri_VSV_ZS_D90 (F2.0).

COMPUTE RQ_Pri_VSV_ZS_D90 =$SYSMIS.

IF ((RQ_Pri_ZS_D90-RQ_Pri_ZS_B) >=RQ_Pri_MID_ZS) RQ_Pri_VSV_ZS_D90 =2.

IF ((RQ_Pri_ZS_D90-RQ_Pri_ZS_B) <RQ_Pri_MID_ZS) RQ_Pri_VSV_ZS_D90 =1.

VARIABLE LABELS RQ_Pri_VSV_ZS_D90 'Relevant verschil tussen vsvs D90- pri score'.

VALUE LABELS RQ_Pri_VSV_ZS_D90

1 'no relevant difference'

2 'relevant difference'.

FREQUENCIES RQ_Pri_VSV_ZS_D90.

NUMERIC RQ_Com_VSV_ZS_D90 (F2.0).

COMPUTE RQ_Com_VSV_ZS_D90 =$SYSMIS.

IF ((RQ_Com_ZS_D90-RQ_Com_ZS_B) >=RQ_Com_MID_ZS) RQ_Com_VSV_ZS_D90 =2.

IF ((RQ_Com_ZS_D90-RQ_Com_ZS_B) <RQ_Com_MID_ZS) RQ_Com_VSV_ZS_D90 =1.

VARIABLE LABELS RQ_Com_VSV_ZS_D90 'Relevant verschil tussen vsvs D90- com score'.

VALUE LABELS RQ_Com_VSV_ZS_D90

1 'no relevant difference'

2 'relevant difference'.

FREQUENCIES RQ_Com_VSV_ZS_D90.

NUMERIC RQ_Tyd_VSV_ZS_D90 (F2.0).

COMPUTE RQ_Tyd_VSV_ZS_D90 =$SYSMIS.

IF ((RQ_Tyd_ZS_D90-RQ_Tyd_ZS_B) >=RQ_Tyd_MID_ZS) RQ_Tyd_VSV_ZS_D90 =2.

IF ((RQ_Tyd_ZS_D90-RQ_Tyd_ZS_B) <RQ_Tyd_MID_ZS) RQ_Tyd_VSV_ZS_D90 =1.

VARIABLE LABELS RQ_Tyd_VSV_ZS_D90 'Relevant verschil tussen vsvs D90- tijd score'.

VALUE LABELS RQ_Tyd_VSV_ZS_D90

1 'no relevant difference'

2 'relevant difference'.

FREQUENCIES RQ_Tyd_VSV_ZS_D90.

NUMERIC RQ_Soc_VSV_ZS_D90 (F2.0).

COMPUTE RQ_Soc_VSV_ZS_D90 =$SYSMIS.

IF ((RQ_Soc_ZS_D90-RQ_Soc_ZS_B) >=RQ_Soc_MID_ZS) RQ_Soc_VSV_ZS_D90 =2.

IF ((RQ_Soc_ZS_D90-RQ_Soc_ZS_B) <RQ_Soc_MID_ZS) RQ_Soc_VSV_ZS_D90 =1.

VARIABLE LABELS RQ_Soc_VSV_ZS_D90 'Relevant verschil tussen vsvs D90- soc score'.

VALUE LABELS RQ_Soc_VSV_ZS_D90

1 'no relevant difference'

2 'relevant difference'.

FREQUENCIES RQ_Soc_VSV_ZS_D90.

NUMERIC RQ_Fac_VSV_ZS_D90 (F2.0).

COMPUTE RQ_Fac_VSV_ZS_D90 =$SYSMIS.

IF ((RQ_Fac_ZS_D90-RQ_Fac_ZS_B) >=RQ_Fac_MID_ZS) RQ_Fac_VSV_ZS_D90 =2.

IF ((RQ_Fac_ZS_D90-RQ_Fac_ZS_B) <RQ_Fac_MID_ZS) RQ_Fac_VSV_ZS_D90 =1.

VARIABLE LABELS RQ_Fac_VSV_ZS_D90 'Relevant verschil tussen vsvs D90- fac score'.

VALUE LABELS RQ_Fac_VSV_ZS_D90

1 'no relevant difference'

2 'relevant difference'.

FREQUENCIES RQ_Fac_VSV_ZS_D90.

NUMERIC RQ_Keu_VSV_ZS_D90 (F2.0).

COMPUTE RQ_Keu_VSV_ZS_D90 =$SYSMIS.

IF ((RQ_Keu_ZS_D90-RQ_Keu_ZS_B) >=RQ_Keu_MID_ZS) RQ_Keu_VSV_ZS_D90 =2.

IF ((RQ_Keu_ZS_D90-RQ_Keu_ZS_B) <RQ_Keu_MID_ZS) RQ_Keu_VSV_ZS_D90 =1.

VARIABLE LABELS RQ_Keu_VSV_ZS_D90 'Relevant verschil tussen vsvs D90- keu score'.

VALUE LABELS RQ_Keu_VSV_ZS_D90

1 'no relevant difference'

2 'relevant difference'.

FREQUENCIES RQ_Keu_VSV_ZS_D90.

## Profiling units performed in SPSS

NUMERIC F_VSV_19 (F2.0).

COMPUTE F_VSV_19 = $SYSMIS.

IF (vsv=19) F_VSV_19 =1.

VARIABLE LABELS F_VSV_19 'Filter best performing unit'.

FREQUENCIES F_VSV_19.

NUMERIC F_VSV_2 (F2.0).

COMPUTE F_VSV_2 = $SYSMIS.

IF (vsv=2) F_VSV_2 =1.

VARIABLE LABELS F_VSV_2 'Filter worst performing unit (2)'.

FREQUENCIES F_VSV_2.

NUMERIC F_VSV_34 (F2.0).

COMPUTE F_VSV_34 = $SYSMIS.

IF (vsv=34) F_VSV_34 =1.

VARIABLE LABELS F_VSV_34 'Filter worst performing unit (34)'.

FREQUENCIES F_VSV_34.

NUMERIC F_VSV_63 (F2.0).

COMPUTE F_VSV_63 = $SYSMIS.

IF (vsv=63) F_VSV_63 =1.

VARIABLE LABELS F_VSV_63 'Filter worst performing unit (63)'.

FREQUENCIES F_VSV_63.

NUMERIC F_VSV_72 (F2.0).

COMPUTE F_VSV_72 = $SYSMIS.

IF (vsv=72) F_VSV_72 =1.

VARIABLE LABELS F_VSV_72 'Filter worst performing unit (72)'.

FREQUENCIES F_VSV_72.

SORT CASES BY F_VSV_19.

FILTER BY F_VSV_19.

FREQUENCIES VARIABLES=RQ_R_Dom_A RQ_A_Dom_A RQ_P_Dom_A RQ_C_Dom_A RQ_T_Dom_A RQ_S_Dom_A RQ_F_Dom_A RQ_K_Dom_A

/STATISTICS=MEAN MEDIAN

/ORDER=ANALYSIS.

FREQUENCIES RQ_C_Ant_A_M RQ_C_Adv_A_M RQ_C_Uit_A_M RQ_C_Inf_A_M.

FILTER OFF.

SORT CASES BY F_VSV_2.

FILTER BY F_VSV_2.

FREQUENCIES VARIABLES=RQ_R_Dom_A RQ_A_Dom_A RQ_P_Dom_A RQ_C_Dom_A RQ_T_Dom_A RQ_S_Dom_A RQ_F_Dom_A RQ_K_Dom_A

/STATISTICS=MEAN MEDIAN

/ORDER=ANALYSIS.

FREQUENCIES RQ_C_Ant_A_M RQ_C_Adv_A_M RQ_C_Uit_A_M RQ_C_Inf_A_M.

FILTER OFF.

SORT CASES BY F_VSV_72.

FILTER BY F_VSV_72.

FREQUENCIES VARIABLES=RQ_R_Dom_A RQ_A_Dom_A RQ_P_Dom_A RQ_C_Dom_A RQ_T_Dom_A RQ_S_Dom_A RQ_F_Dom_A RQ_K_Dom_A

/STATISTICS=MEAN MEDIAN

/ORDER=ANALYSIS.

FREQUENCIES RQ_C_Ant_A_M RQ_C_Adv_A_M RQ_C_Uit_A_M RQ_C_Inf_A_M.

FILTER OFF.

SORT CASES BY F_VSV_34.

FILTER BY F_VSV_34.

FREQUENCIES VARIABLES=RQ_R_Dom_A RQ_A_Dom_A RQ_P_Dom_A RQ_C_Dom_A RQ_T_Dom_A RQ_S_Dom_A RQ_F_Dom_A RQ_K_Dom_A

/STATISTICS=MEAN MEDIAN

/ORDER=ANALYSIS.

FREQUENCIES RQ_C_Ant_A_M RQ_C_Adv_A_M RQ_C_Uit_A_M RQ_C_Inf_A_M.

FILTER OFF.

SORT CASES BY F_VSV_63.

FILTER BY F_VSV_63.

FREQUENCIES VARIABLES=RQ_R_Dom_A RQ_A_Dom_A RQ_P_Dom_A RQ_C_Dom_A RQ_T_Dom_A RQ_S_Dom_A RQ_F_Dom_A RQ_K_Dom_A

/STATISTICS=MEAN MEDIAN

/ORDER=ANALYSIS.

FREQUENCIES RQ_C_Ant_A_M RQ_C_Adv_A_M RQ_C_Uit_A_M RQ_C_Inf_A_M.

FILTER OFF.
