# Supplementary material for: The discriminative power of the ReproQ: a client experience questionnaire in maternity care
Source: PeerJ. 2019 Nov 27;7:e7575. doi: 10.7717/peerj.7575 (PMC6884994; doi:10.7717/peerj.7575)
Supplement: Supplemental Information 4 [file peerj-07-7575-s004.docx]

**TECHNICAL SUPPLEMENT**
Differences in summary ReproQ scores across perinatal units, the dependent variable, may originate from three sources: 1) ‘true’ differences across perinatal units, 2) differences in client characteristics across perinatal units, 3) residual variance. Given the hierarchical data structure (perinatal units and clients within perinatal units), existing differences in client characteristics across perinatal units may obscure the estimation of the ‘true’ difference across units. When data are hierarchically ordered, multilevel analysis rather than conventional ordinary linear regression analysis is the appropriate analytical framework ([Twisk 2014](#_ENREF_11)). Multi-level analysis decomposes total data variance into variance attributable to perinatal units (source 1) and variance attributable to other sources (sources 2+3), in particular variance related to client characteristics. Estimation of the ‘true’ difference between perinatal units (source 1) requires that the domain and summary scores should be corrected for the other variance components (typically client characteristics) that bias and limit the comparison of perinatal units (i.e. the case mix correction). The need for case mix correction may be judged from the extent to which the clients’ characteristics are related to the domain and summary scores of the perinatal unit they received care in, using Intraclass Correlation Coefficients (ICCs). The comprehensive analysis of variance components and ICCs is called a generalizability or G-study ([Streiner et al. 2014](#_ENREF_9)). The methodology has been described and applied in other client experiences measurement studies as well ([Bos et al. 2015](#_ENREF_2); [Stubbe et al. 2007](#_ENREF_10)). A key result of multilevel analysis is the point estimate (and 95% confidence interval [CIs]) of the ‘true’ perinatal unit effect (one estimate for each perinatal unit) corrected for case mix differences. Note that the size of the 95%CIs differs across perinatal units, reflecting (a) different numbers of clients across perinatal units, and (b) different degrees of heterogeneity of client characteristics across perinatal units.

*Case mix correction*

The conventional procedure to determine the need for *case mix correction* is to compare the results of two multilevel regression models: (1) an ‘empty’ model with the ReproQ domain or summary scores as dependent variable, and solely a random intercept for each perinatal unit; and (2) an corrected model, with the ReproQ domain or summary score as dependent variable, a random intercept for each perinatal unit, and client characteristics included as explanatory variables ([Bos et al. 2015](#_ENREF_2); [Stubbe et al. 2007](#_ENREF_10)).
We avoided overfitting and overcorrection, by limiting correction to predefined candidate factors with a demonstrated effect. Of the available client characteristics (age, educational level, ethnicity, parity, and self-rated health) only age, educational level, and self-rated health contributed significantly (p<0.05) to all domain and summary scores, and were therefore were included in the case mix correction. We also tested for random slopes, but none of these were significant and therefore remain unreported.

*Discriminative power: Two approaches*

In our study design, groups of individual clients’ scores are associated with (nested within) perinatal units. Although some of the variance in clients’ scores can be attributed to individual experiences, some of the variance in clients’ scores is likely to be attributable to perinatal unit, with some perinatal units performing better than others ([Roberts et al. 2014](#_ENREF_4)). In our analyses we corrected for this nested structure.

Next, we used two complementary approaches to determine discriminative power.

*Approach 1. Multilevel testing of the deviation of* unit *means from overall mean*

Multi-level analyses were used to examine to what degree ReproQ is able to identify units that significantly perform above and below average (averaged over perinatal units), producing three parts of information: 1) the estimation of variance components and ICCs; 2) the estimation of the G-coefficient; and 3) the resulting estimation of the number of clients needed per perinatal unit to achieve sufficient reliability (D-Study). The conventional mode of presentation is the so-called caterpillar-plot.

In the first part, the output of the multilevel analysis in terms of variance components (explanatory variables) is used, and intra-class correlation coefficients (ICCs) are computed using these quantities. Here, the ICC of interest is the ratio of the variance in perinatal unit and the variance in client’s characteristics in that unit ([Streiner & Norman 2008](#_ENREF_8); [Streiner et al. 2014](#_ENREF_9)). An ICC close to zero implies that the client’s experience is unrelated to the perinatal unit in which one receives care. In that case benchmarking of perinatal units with ReproQ would be of no value. In contrast when the ICC is close to 1, the perinatal unit is of decisive importance. The ICC-calculations were performed on the empty model as well as the corrected model (see before). The same output is used to obtain uncorrected and corrected means per perinatal unit, with their 95% confidence intervals. Best and poor practices are identified by the deviation of the 95% CI of each individual perinatal unit from the grand mean of all perinatal units (above or below the grand mean). Unit mean, their CIs, and the grand mean are conventionally plotted as caterpillar plots, ranking the perinatal units according to each unit’s deviation from the grand mean domain or summary score (see figure 1 for an example). Note that the grand mean is a common, yet arbitrary reference or norm.

In the second part, the reliability of the perinatal unit effect is established, given the realized numbers of clients; this is called the G-coefficient ([Streiner et al. 2014](#_ENREF_9)). The G-coefficient expresses the proportion of variance in perinatal unit-level mean scores attributable to ‘true’ variation among perinatal units. The G refers to 'generalizability theory' ([Shavelson et al. 1989](#_ENREF_6); [Streiner et al. 2014](#_ENREF_9)). Generalizability theory is a conventional base to study nested psychological data, like here. It assumes that any measurement is subject to multiple sources of error variance, such as client characteristics. A G-coefficient of one (the highest possible score) implies that all variance in domain and summary scores across perinatal units is attributed to the perinatal unit, and no variance can be attributed to other sources.

The third part, calculated for the average perinatal unit, and for a predefined level of reliability (more certainty requires more respondents) the minimum number of clients per perinatal unit to achieve significance. Small numbers of clients and large heterogeneity of client experiences produce wide confidence intervals, where only the first can be influenced. We deliberately selected units with at least 50 clients, but sample size still differed considerably across units as did the heterogeneity of their experiences. The aim of a D-Study (D from ‘decision’) is to estimate the number of included clients that is needed to achieve for any perinatal unit a predefined level of reliability, usually 0.80([Shavelson et al. 1989](#_ENREF_6); [Streiner et al. 2014](#_ENREF_9)). Stated otherwise, a D-Study estimates the minimal number of clients needed to create sufficient discriminative power for benchmark purposes, by using the variances derived from the G-study.

*Approach 2. Relevance deviation based on MID*

This approach judges ReproQ’s discriminative power on the basis of the ability to proof relevant differences in domain or summary scores at the perinatal unit level; we introduced the MID for this purpose. We were aware that a MID at the individual level is not the same as the MID at the unit level (see below).
We previously determined, at the individual level, the minimally important difference (MID) for both the summary score and the individual domain scores, for the childbirth phase only ([Scheerhagen et al. 2016](#_ENREF_5)). The distribution-based and anchor point based MID had comparable size. For this paper, we derived MIDs for the late pregnancy and postnatal period in a similar way, see table 2.
While a MID of 1 unit by definition is relevant if individual client’s scores are compared with a norm or with each other, 1 MID difference at the perinatal unit level reflects a large difference: it means that *all* clients cared for in that unit, on average differ 1 MID from a reference value, either being much better (best practice) or much worse (poor practice). Compare birth weight, where a 200 gram difference of an individual from the gestational age norm is judged trivial, while an average 200 gram difference represents an important difference at the group level. We therefore added as a second criterion a difference of 0.5 MID unit, which still represents a stringent relevance criterion at the organisational level. As a norm to compare unit averages (summary score, domain scores) with in terms of the MID, we used the pooled average domain and summary scores of the 10% best performing units.

*Statistical software*
We computed all multi-level analyses and ICCs with R version 3.2.3 ([R Core Team 2015](#_ENREF_3)), using the ggPlot program for graphical display of output, in particular an adapted version of the ggCaterpillar function for creating the caterpillar plots ([StackOverflow 2013](#_ENREF_7)). G-coefficients and D-sample sizes were estimated using G_string_IV, using the so-called ‘unbalanced, nested one facet-design’ (design 1.3) with perinatal unit as cluster [nesting variable] and client as facet ([Bloch & Norman 2015](#_ENREF_1)). (Details can be obtained from the authors.) For other statistical analyses we used SPSS 20.0.

**References**

Bloch R, and Norman G. 2015. G String IV. Hamilto, Canada.

Bos N, Sturms LM, Stellato RK, Schrijvers AJ, and van Stel HF. 2015. The Consumer Quality Index in an accident and emergency department: internal consistency, validity and discriminative capacity. *Health Expect* 18:1426-1438.

R Core Team. 2015. R: A Language and Environment for Statistical Computing. Vienna, Austria: R Foundation for Statistical Computing.

Roberts MJ, Campbell JL, Abel GA, Davey AF, Elmore NL, Maramba I, Carter M, Elliott MN, Roland MO, and Burt JA. 2014. Understanding high and low patient experience scores in primary care: analysis of patients' survey data for general practices and individual doctors. *Bmj* 349:g6034.

Scheerhagen M, van Stel HF, Tholhuijsen DJ, Birnie E, Franx A, and Bonsel GJ. 2016. Applicability of the ReproQ client experiences questionnaire for quality improvement in maternity care. *PeerJ* 4:e2092. 10.7717/peerj.2092

Shavelson RJ, Webb NM, and Rowley GL. 1989. Generalizability theory. *American Psychologist* 44:922-932.

StackOverflow. 2013. In R plotting random effects. *Available at* <https://stackoverflow.com/questions/13847936/in-r-plotting-random-effects-from-lmer-lme4-package-using-qqmath-or-dotplot>.

Streiner DL, and Norman GR. 2008. *Health measurement scales*. Oxford: University Press.

Streiner DL, Norman GR, and Cairney J. 2014. *Health measurement scales*. Oxford: University Press.

Stubbe JH, Brouwer W, and Delnoij DM. 2007. Patients' experiences with quality of hospital care: the Consumer Quality Index Cataract Questionnaire. *BMC Ophthalmol* 7:14.

Twisk JWR. 2014. *Applied Multilevel Analysis*: Cambridge University Press.
